# Tobacco and Alcohol Consumption Rates among Chinese Women of Reproductive Age in 2004–2011: Rate and Sociodemographic Influencing Factors

**DOI:** 10.3390/ijerph16010056

**Published:** 2018-12-26

**Authors:** Ruiyi Liu, Li Chen, Huan Zeng, Cesar Reis, Haley Reis, Xianjie Yang, Xinjie Lin, Huabing Li, Xuchen Meng, Manoj Sharma, Yong Zhao

**Affiliations:** 1 School of Public Health and Management, Chongqing Medical University, Chongqing 400016, China; 2016223483@stu.cqmu.edu.cn (R.L.); 2015221713@stu.cqmu.edu.cn (L.C.); zenghuan586@aliyun.com (H.Z.); 2017222982@stu.cqmu.edu.cn (H.L.); 2 Research Center for Medicine and Social Development, Chongqing Medical University, Chongqing 400016, China; 3 The Innovation Center for Social Risk Governance in Health, Chongqing Medical University, Chongqing 400016, China; 4 Preventive and Occupational Medicine Department, Loma Linda University Medical Center, Loma Linda, CA 92354, USA; creis@llu.edu; 5 School of Medicine, Loma Linda University, Loma Linda, CA 92354, USA; hreis@llu.edu; 6 Clinical College, Chongqing Medical University, Chongqing 400016, China; 2015221089@stu.cqmu.edu.cn (X.Y.); 2017222992@stu.cqmu.edu.cn (X.L.); 2017223007@stu.cqmu.edu.cn (X.M.); 7 Department of Behavioral and Environmental Health, Jackson State University, Jackson, MS 39213, USA; manoj.sharma@jsums.edu

**Keywords:** Chinese women, tobacco consumption, alcohol consumption, reproductive stages

## Abstract

*Background*: Smoking and alcohol consumption have become major public health problems among Chinese women. In this study we explore the behavioral trends in smoking and alcohol consumption of Chinese women. We also explored the changes in the sociodemographic factors that affect the smoking and alcohol consumption behaviors of Chinese women at different reproductive stages. *Methods*: We used the Chinese Health and Nutrition Survey data for 2004 to 2011 to investigate the trends and influential factors of tobacco and alcohol consumption among Chinese women. Data for tobacco and alcohol consumption (consumption of beer or any other alcoholic beverage and smoking of cigarettes) were extracted using questionnaires. We applied the *χ^2^* test to examine the trends of alcohol and tobacco consumption among Chinese women over the period of 2004 to 2011. We conducted two penalized logistic regressions with age as the continuous and classification variable (18–23, 24–29, 30–44, and 45–49 years), and independent variables included residence, age, and marital status. *Results*: Drinking rates among Chinese women significantly changed over the period of 2004 to 2011 (*p* = 0.018). Age was related to tobacco consumption rates for 2009 and 2011 (*p* < 0.05). Marital status was associated with tobacco consumption rates for 2004, 2009, and 2011 (*p* < 0.05). Tobacco and alcohol consumption rates from 2004 to 2011 were positively correlated (*p* < 0.05). Over the period of 2004 to 2011, alcohol consumption rates were higher among women living in urban areas than those among women living in rural areas (*p* < 0.05). High educational attainment was related to alcohol consumption. Educational attainment levels of secondary or primary schooling and university or above were related to alcohol consumption rates for 2004 to 2011 (*p* < 0.05). Employed women were more likely to consume alcohol than unemployed women in 2004, 2006, and 2011 (*p* < 0.05). Data from 2004 to 2011 showed that tobacco and alcohol use were correlated (*p* < 0.05) and that women aged 45–49 years old were more likely to consume tobacco than other women (*p* < 0.05); *Conclusions*: The drinking behavior of Chinese women changed considerably over the period of 2004 to 2011. Our results provide further insight on the smoking and drinking behaviors of Chinese women at different reproductive stages and the factors that influence such behaviors. Therefore, our findings on trends and factors that influence rates of tobacco and alcohol use allow for a better understanding of the smoking and drinking behaviors of Chinese women.

## 1. Introduction

Tobacco and alcohol use causes serious public health problems among women worldwide. In 2010, the percentage of female smokers worldwide reached 9%, and that of female smokers in China reached 2.5%. The percentage of female smokers in the Chinese population is lower than the global rates. Nevertheless, given that women account for 600 million of the total Chinese population, the rates of smoking among women in China warrants discussion. Despite the institution of a series of tobacco control measures, 2.7% of women in China reported having consumed tobacco in 2015 [1]. In addition, the rates of tobacco consumption are increasing among Chinese women. Additional research is needed to understand the trends and influential factors of tobacco consumption among Chinese women.

Reports by the World Health Organization (WHO) stated that the harmful effects of alcohol consumption cause 2.5 million deaths per year [2]. Women are highly vulnerable to the harmful effects of alcohol. In 2010, women consumed 8.9 L of pure alcohol per capita worldwide. Alcohol-related deaths among women account for 4.0% of all global deaths [3]. In 2010, 14.5% of Chinese women aged 18 years and above consumed alcohol. A 2018 WHO report stated that alcoholic liver cirrhosis accounted for 5.8% of deaths among Chinese women.

Drinking has become increasingly widespread with the improvement in the economic and social status of Chinese women. Thus, studies describing the trends and factors influencing alcohol and tobacco consumption among Chinese women are needed.

Tobacco consumption imposes a major health burden on women. Female smokers are more vulnerable to the harmful effects of smoking than male smokers. For example, female smokers are more likely to develop small-cell carcinoma than male smokers [4,5]. Smoking can cause menstrual disorders and infertility in nonpregnant women [6,7]. In addition, smoking during pregnancy can result in birth defects, and can increase the risk of pregnancy complications, such as miscarriage, premature rupture of membranes, and placenta previa [8,9,10,11]. Smoking can cause early menopause, and doubles the risk of osteoporosis among menopausal women [9,12]. Furthermore, exposure to second-hand smoke contributes to health problems among women. Increased exposure to second-hand smoke is associated with an increased risk of death from cardiovascular disorders [13].

Some of the negative effects of alcohol consumption are more pronounced in women than in men, and women are more likely to suffer from physical and sexual abuse than men when using alcohol. Heavy drinking exerts more negative effects on women’s health than on men’s health, and women are more likely to develop alcohol-related illnesses than men [14]. Female drinkers have a higher risk of breast cancer than female nondrinkers [15]. Drinking during pregnancy can lead to spontaneous abortion, and may have negative effects on the intelligence, memory, and neurodevelopment of children [16]. Alcohol consumption is associated with coronary heart disease and osteoporosis among postmenopausal women [17].

Several studies have explored the sociodemographic factors that affect tobacco and alcohol consumption among women. Age, educational level, and marital status affect tobacco consumption among female Chinese smokers. A study in China found a positive correlation between age and smoking in women [18]. Before 2009, low tobacco consumption rates are associated with high educational attainment [18]. Divorcees or widows are more likely to smoke than other women [19]. In addition, regional location and educational attainment affect alcohol consumption rates among female Chinese drinkers. Specifically, in China, women living in urban areas, and those with a higher level of education have increased alcohol consumption rates compared to their counterparts [20,21].

The smoking and drinking behaviors of women are closely related [21]. Alcohol use increases the risk of exposure to drugs, such as tobacco [22]. The historically low smoking and drinking rates among Chinese women may be attributed to cultural constraints. In recent years however, tobacco and alcohol consumption by young Chinese women has increased. Previous studies examined the trends of alcohol consumption among Chinese women from 1993 to 2006 by using data from the China Health and Nutrition Survey (CHNS) [20]. However, recent trends in alcohol and tobacco consumption among Chinese women have not yet been subjected to multifactorial analysis.

In the present study, we explored the smoking and drinking behaviors of Chinese women by using CHNS data from the years of 2004 to 2011. We conducted a multifactorial analysis, using set inclusion and exclusion criteria to include additional information, such as demographic characteristics. Finally, we discussed the relationship between smoking and drinking among Chinese women. We also considered the differences in smoking and drinking behaviors among women at different reproductive stages. This study specifically aimed to (1) describe the demographic factors and changes that affect the smoking and drinking behaviors of Chinese women; (2) explore the changes in the trends of the smoking and drinking behaviors of Chinese women over the period of 2004 to 2011; and (3) explore the differences in smoking and drinking behaviors of Chinese women at different reproductive stages.

## 2. Materials and Methods

### 2.1. Study Sample and Ethical Considerations

Given that this study used public data collected through the CHNS, our organization did not conduct an ethics review. The CHNS collected data from nine provinces (Liaoning, Jiangsu, Shandong, Henan, Hubei, Hunan, Guangxi, Guizhou, and Heilongjiang) and three major cities (Beijing, Shanghai, and Chongqing). These cities were only included in the CHNS in 2011. Numerous important studies on health, demographic, socioeconomic, and nutritional policies have been conducted on the basis of CHNS data. The present study was based on the CHNS data for 2004, 2006, 2009, and 2011. Missing values were deleted, and the data of 11,236 (2764 in 2004, 2645 in 2006, 2550 in 2009, and 3277 in 2011) adult women (aged 18–49 years) were included. Additional information has been provided in a previous study [23].

### 2.2. Outcome Variables

The survey questionnaire determined alcohol consumption on the basis of the response to the question “Did you drink beer or any other alcoholic beverage this year?” Tobacco consumption was determined on the basis of the response to the question “Have you ever smoked cigarettes (including hand-rolled or device-rolled cigarettes)?” The answers to these questions were either “yes” or “no”. In accordance with the survey questionnaire, we defined drinking as having had at least one drinking experience in the year preceding the survey year, and smoking as having at least one smoking experience. All adults aged 18 years and older were asked to answer the questionnaire.

### 2.3. Covariates

The analysis included the following variables: residence, age, family living situation, marital status, household registration type, highest educational level, current educational status, and work status. Age was divided into four groups, in accordance with the different reproductive stages of women: 18–23 (puberty), 24–29 (optimal reproductive age), 30–44 (premenopausal), and 45–49 years (menopausal). Residence was classified as either urban or rural. The family living situation was divided into either “father/mother resides with family” or “father/mother does not reside with family.” The household registration of the participants was classified as either urban or rural. Educational levels were determined on the basis of the responses to the following two questions: “How many years of formal education have you completed in a regular school?” and “What is the highest level of education you have attained?” The participants were divided into four groups, in accordance with educational attainment: primary school and below, secondary and primary school graduates, high school and secondary technical school, and university or above. The current educational status was determined.

### 2.4. Statistical Analyses

Frequencies and percentages were used to describe the characteristics of the participants. The age of the participants was presented as the mean (standard deviation). The *χ*^2^ test was used to examine the trends in alcohol and tobacco consumption among women during the years of 2004 to 2011. Given that Chinese women rarely smoke or drink [24], alcohol use and tobacco use were used as dependent variables for punishment logistic regression modeling, which is more suitable for our research than the ordinary regression method. Age was included as a continuous variable. Participants were allocated into four groups on the basis of age, to examine the tobacco and alcohol consumption behaviors of adult women at different reproductive stages. Two penalized logistic regression models were developed by using residence and age as classification, and continuous variables and family living situation, marital status, household registration type, highest educational level, current educational status, and work status as independent variables. The models were then used to explore the differences in the smoking and alcohol consumption behaviors of Chinese women at different reproductive stages. All tests were two-sided, and *p* < 0.05 was considered to be statistically significant. All data analyses were performed by using statistical software (SAS version 9.4.3; SAS Institute, Cary, NC, USA).

## 3. Results

### 3.1. Characteristics of the Study Sample

Our study included 11,236 adult women aged 18–49 years old. All participants were identified by using CHNS data. A total of 169 participants reported that they had previous smoking experience, and 1170 participants reported that they had previous drinking experience. Among the participants, 65.02% resided in rural areas. The average age of the participants was 36.96 ± 8.35 years, and 87.78% of the participants were married. Moreover, 59.02% of the participants were from rural households, 67.07% had the highest educational attainment of primary and secondary school or lower, and 70.02% were employed (Table 1).

### 3.2. Trends of Tobacco and Alcohol Consumption Rates

We found that 1.45%, 1.78%, 1.33%, and 1.46% of the recruited participants identified as smokers in 2004, 2006, 2009, and 2011, respectively. In addition, 13.39%, 8.39%, 8.51%, and 11.02% of the participants reported having had previous drinking experiences in 2004, 2006, 2009, and 2011, respectively. The trends of tobacco consumption negligibly differed on a yearly basis. By contrast, the trends of alcohol consumption significantly differed (*p* = 0.018) on a yearly basis. Drinking rates fell sharply in 2006 and 2009, but rose again in 2011 (Table 2).

### 3.3. Penalized Logistic Regression Analysis of Tobacco Consumption among Participants without Age Classification

Our data showed that age was positively correlated with tobacco consumption among adult women in 2009 and 2011. In 2004, 2009, and 2011, married participants were less likely to smoke than single women. In 2004, 2006, 2009, and 2011, adult women with previous drinking experience were more likely to smoke than those without previous drinking experience (Table 3).

### 3.4. Penalized Logistic Regression Analysis of Alcohol Consumption among Participants without Age Classification

From 2004 to 2011, respondents who resided in rural areas were less likely to drink than those who resided in urban areas. Secondary and primary school graduates were less likely to drink than those with an educational level of primary school or below. Alcohol consumption rates increased among participants with an educational attainment of university or above, but remained constant among women with an educational attainment of high school and secondary technical school. In 2004, 2006, and 2011, unemployed respondents were less likely to drink than employed respondents. In 2004, 2006, 2009, and 2011, women with previous smoking experience were more likely to drink than those without prior smoking experience (Table 4).

### 3.5. Penalized Logistic Regression Analysis of Tobacco Consumption among Participants with Age Classification

In 2004 and 2011, respondents aged 30–44 years old were more inclined to smoke than those aged 18–23 years old. In 2004, 2006, 2009, and 2011, respondents aged 45–49 years old were more inclined to smoke than those aged 18–23 years old. In 2004, 2009, and 2011, married women were less likely to smoke than single women. In 2004 and 2006, respondents from rural households were more likely to smoke than those from urban households. Adult women with previous drinking experience were more likely to smoke than those without previous drinking experience (Table 5).

### 3.6. Penalized Logistic Regression Analysis of Alcohol Consumption among Participants with Age Classification

From 2004 to 2011, respondents from rural areas were less inclined to drink than those from urban areas. In 2004, respondents aged 45–49 years old were more likely to drink than those aged 18–23 years old. In 2004, 2006, 2009, and 2011, respondents with an educational attainment of secondary and primary school were less likely to drink than those with an educational attainment of primary school or below. In 2004 and 2009, respondents with an educational attainment of high school and secondary technical school were likely to drink. Similarly, in 2004, 2006, 2009, and 2011, adult women with an educational attainment of university or above were likely to drink. In 2004, 2006, and 2011, unemployed respondents were less likely to drink than employed respondents. In 2004 to 2011, drinking was more widespread among adult women with previous smoking experience than those without previous smoking experience (Table 6).

Overall, we found similar results for tobacco consumption (Table 3 and Table 5). Age, marital status, and alcohol consumption were considerably correlated with tobacco consumption among adult women of reproductive age. In addition, residence, high educational attainment, working status, and tobacco consumption were considerably associated with alcohol consumption (Table 4 and Table 6). Women in different age groups exhibited different smoking or drinking tendencies (Table 5 and Table 6). In 2004 and 2011, women aged 30–44 years old were more likely to smoke than those aged 18–23 years old. In 2004, 2006, 2009, and 2011, women aged 45–49 years old were more likely to smoke than those aged 18–23 years old. In 2004, women aged 45–49 years old were more likely to drink than those aged 18–23 years old.

We found that observations were low for those divorced, single, widowed, and separated women. We eliminated those classifications, ran the model using the data for currently married or unmarried women, and subjected the sample to penalized logistic regression analysis. In 2009 and 2011, married participants were less likely to smoke than unmarried women. The alcohol consumption rates of married and unmarried respondents for 2004, 2006, 2009, and 2011 did not significantly differ (Tables S1–S3 in Supplementary File).

## 4. Discussion

We investigated the trends and influencing factors of smoking and alcohol consumption among Chinese women at different reproductive stages (puberty: 18–23 years, optimal reproductive age: 24–29 years, premenopausal: 30–44 years; and menopausal: 45–49 years). In 2004, 2006, 2009, and 2011, women aged 45–49 years old tended to smoke more than women aged 18–23 years old. In 2004, women aged 45–49 years old tended to drink more than women aged 18–23 years old. Furthermore, we found that alcohol consumption rates among Chinese women drastically changed in 2004, 2006, 2009, and 2011.

We found that alcohol consumption rates among Chinese women dropped sharply from 2004 to 2006, and began to increase gradually from 2006 to 2011. However, alcohol consumption rates for 2011 remained lower than those for 2004. Similar to our study, a previous study found that alcohol consumption rates among Chinese women gradually declined from 2004 to 2006 [20]. This reduction may be attributed to the close relationship between the changes in women’s drinking rates and economic changes. First, nationwide real estate sales rose by 9.7% in 2004 relative to those in 2003. In Chinese traditional culture, the house is the symbol of the family. Rapid increases in housing prices deterred most families from purchasing houses. Increased housing demands increased the stress experienced by families. Women, in particular, bear the brunt of the stress experienced by families. At the same time, the purchasing power of Chinese women remained strong, given that China’s economy was still rapidly developing. Many women consumed alcohol to relieve stress. Thus, alcohol consumption rates remained high in 2004. From 2004 to 2006, however, China experienced a stock market crash, and major food safety problems. The stock market crash drastically reduced the purchasing power of women in China. In addition, several incidences of the adulteration of edible alcohol with industrial alcohol aroused public distrust in alcoholic drinks. As a result, drinking rates among Chinese women fell sharply between 2004 and 2006. From 2006 to 2011, China’s economy entered a stable state of growth, and gradually recovered from the effects of the stock market crash. In addition, the Chinese government introduced a series of policies to ensure the safety of alcohol products. The alcohol consumption rate among Chinese women increased gradually over this period, and gradually recovered. Nevertheless, the alcohol consumption rate for 2011 remained lower than that for 2004, because women had gained a good understanding of the negative effects of alcohol. The alcohol consumption rates obtained by our study may be lower than the actual rates. This inconsistency may be attributed to study limitations or to the differences in cultural background, economic level, social status [25], and educational levels among women in different regions.

Similar to previous studies [26], our study showed that mature married women were less likely to smoke than young single women. Smoking rates among Chinese men are some of the highest worldwide. The smoking behaviors of married women may be influenced by those of their partners. Partners play an active role in the decision and behavior of women to quit smoking [27]. In addition, most married women are pregnant, or have already become pregnant. Numerous studies have demonstrated the adverse effects of smoking on pregnant women and infants. Thus, husbands and partners actively support and encourage their wives and partners to quit smoking. Nevertheless, the smoking behavior of partners is a risk factor for smoking among pregnant women [28]. Community health education can help raise awareness on the dangers of tobacco use among pregnant women, and effectively encourage women to quit smoking [29]. Smoking rates among women increase with age. Thus, different health education strategies that promote smoking cessation and that target specific age groups of women must be developed. Future research should consider the effects of health education on the decisions of pregnant women and their partners to quit smoking. Numerous public service advertisements regarding tobacco control are televised in China. Many of these advertisements focus on the disadvantages of tobacco exposure during pregnancy. Graphic warnings, particularly advertisements showing the real consequences of smoking, generate strong reactions [30]. The serious adverse effects of smoking on infants are important factors that influence the decision of married women to quit smoking. Public service advertisements and other forms of publicity should emphasize and describe the consequences of smoking, to promote awareness and smoking cessation.

We found that women who reside in rural areas and who are unemployed were less inclined to drink than those who live in urban areas and who are employed. Women living in urban areas are stressed to the point that they experience negative emotions, such as depression and anxiety [31]. A previous study showed that women from rural areas are likely to begin drinking heavily after moving to urban areas, in response to high situational pressure [32]. Women who are affected by negative emotions, such as depression, are likely to drink heavily [33,34]. Heavy drinking is an important predictor of risky behaviors, such as suicide, among women [35]. Meanwhile, different jobs may also increase alcohol consumption in women. Women may engage in male-dominated occupations requiring masculine behavioral patterns that conflict with female behavioral patterns. Hence, women in such occupations may consume alcohol to relieve the negative emotions that result from high personal and societal pressure [36]. Work–family conflicts are also important factors for heavy drinking among women [37]. Excessive drinking can reduce effectiveness at work, and ultimately result in job loss. Unemployment exerts burden on families. Reduced family support can lead to heavy drinking among women [38]. We also found that women with an educational attainment of middle school were less likely to drink alcohol than those with an educational attainment of primary school or below. Women with an educational attainment of university or above, however, were likely to drink. This pattern may be attributed to the engagement of women with high educational levels in socially important work. In some workplaces, women may resort to masculine behaviors, such as heavy drinking, to gain social status. In the future, we can study the coeffects of different jobs and educational levels on women’s drinking behaviors, and establish a sound social mechanism to address the mental health of women. Notably, the amount of alcohol consumption in Chinese women was not included in the study. Additionally, it is possible that some of the women who drink do not reach binge drinking levels, and do not require intervention. However, we also discussed the negative effects of heavy drinking to arouse women’s attention to drinking behaviors and to further promote female abstinence, although the drinking rate may not be high among women.

We found that women aged 45–49 years old were more likely to smoke than adolescents. Previous studies showed that smoking during menopause results in decreased bone mineral density and abnormal BMI [39]. A previous study in China found that menopausal women have poor psychological, social, and physiological qualities of life [40]. These problems make women aware of aging, and they develop negative attitudes. Women with negative attitudes are likely to show symptoms of depression, irritability, and fatigue [41]. Smoking is associated with negative emotions, such as depression, which may be a major cause of smoking problems among menopausal women [42]. A previous research found that Chinese women who lead active lives do not notice their aging. Women in their 40s exhibited the least positive attitude towards aging [41]. This attitude may explain the widespread popularity of physical activities, such as square dancing, among Chinese women. Proper physical activity can improve the quality of life [43], alleviate negative attitudes towards aging, and promote smoking cessation among Chinese women. We also recommend adopting measures to address the psychological problems experienced by Chinese women. While the smoking behaviors of these women may not be sufficiently severe to warrant intervention, we suggest developing measures that might help prevent women from initiating smoking behavior.

Similar to previous studies [21,23], our study found that female drinkers were likely to smoke, and that female smokers were likely to drink. The use of alcohol tends to increase the use of other addictive drugs, such as tobacco [22]. The social status of Chinese women has continuously been improving, and Chinese women actively participate in social occasions. Many women believe that cigarettes and alcohol act as catalysts to liven up the atmosphere at parties, and those who drink in small quantities also begin smoking. In addition, smoking and drinking are widespread among young Chinese women [18,44]. The advertising industry has falsely associated the use of tobacco and alcohol with beauty, charm, and freedom. This false association has prompted young Chinese women to combine tobacco and alcohol consumption.

The WHO stated that compared with Japanese women with similar cultural backgrounds, Chinese women tend to consume less amounts of tobacco but similar amounts of alcohol [45,46]. Japan’s tobacco control policies are effective. The per capita alcohol consumption in China is increasing, whereas that in Japan is declining. Although our study did not focus on the specific amounts of tobacco and alcohol consumed by Chinese women, we examined the rising trend of drinking rates among Chinese women. Given China’s large population, even a low alcohol consumption rate indicates that a large number of women are drinking alcohol. From 2004 to 2011, China underwent rapid economic development, and an increasing number of women began to live independent and free lifestyles. Traditional Chinese culture does not approve of smoking and drinking by women. However, these behaviors have become acceptable, given that Chinese society has become increasingly open and inclusive. At the same time, women have begun to assume different societal roles while bearing most of the responsibilities and pressures in the family. Working women have to use tobacco or alcohol in social situations to improve their relationships, and alcohol seems to be a more popular choice than tobacco.

Our study has several advantages. First, we used data from longitudinal studies conducted by the CHNS, and included data from 2004 to 2011. This approach allowed us to characterize recent trends in the smoking and drinking behaviors of Chinese women. Second, our results reflect the smoking and drinking behaviors of Chinese women, given the large sample size of our study. Third, we focused on the trends shown by the changes in the drinking behavior of Chinese women and the factors that influence their smoking and drinking behaviors at different ages. Our study is essential for determining the changes in the smoking and drinking behaviors of Chinese women of different ages under the influence of sociodemographic factors. However, our study also has several limitations. The subjects of the CHNS survey vary yearly. In addition, the CHNS does not fully reflect the current situation of the smoking and drinking behaviors of Chinese women, because it is not a nationally administered survey. Thus, the CHNS may underestimate smoking and drinking rates among Chinese women, especially the low rate of smoking. Although some measures have been taken to reduce the deviation caused by the low smoking rate, this effect may still exist. Notably, in the CHNS study, some female participants reported consuming tobacco or alcohol, but did not report the amount of tobacco and alcohol that they had consumed. The current study only focused on whether women smoked at least once, and whether they had drunk alcohol in the year preceding the study year, and not the specific amount of tobacco and alcohol that they had consumed. Meanwhile, the CHNS study did not distinguish between women with smoking/ drinking experience, and current or ever smokers/drinkers. In addition, our study only analyzed the data for 2004, 2006, 2009, and 2011. Some influential sociodemographic factors did not show strong correlations in all the survey years. Lastly, we were unable to analyze behavioral trends after 2011, given that the latest data have not been released yet.

## 5. Conclusions

Alcohol consumption rates among Chinese women decreased drastically from 2004 to 2006, and recovered from 2006 to 2011. Nevertheless, the rates of alcohol consumption in 2011 remained lower than those in 2004. Women at different reproductive stages showed drastically different smoking behaviors. The smoking and drinking behaviors of Chinese women over the period of 2004 to 2011 were influenced by social demographic factors, such as age, marital status, and residence. Our results remind us of the necessity of evaluating the psychological status of women aged 45–49 years old, and exploring the social and demographic factors that affect the smoking and drinking behaviors of women at different reproductive stages.

## Figures and Tables

**Table 1:** Characteristics of 11,236 participants stratified by survey year (*N*, %).

Variable		2004	2006	2009	2011	Total	Smoker ^1^	Drinker ^2^
Residence	Urban	928 (33.57)	871 (32.93)	816 (32.00)	1315 (40.13)	3930 (34.98)	62 (36.69)	647 (55.30)
	Rural	1836 (66.43)	1774 (67.07)	1734 (68.00)	1962 (59.87)	7306 (65.02)	107 (63.31)	523 (44.70)
Age		39.04 ± 8.44	37.68 ± 8.15	36.03 ± 8.21	35.34 ± 8.09	36.96 ± 8.35	—	—
Father resides with family	Yes	281 (10.17)	270 (10.21)	273 (10.71)	380 (11.60)	1204 (10.72)	8 (4.73)	146 (12.48)
	No	2483 (89.83)	2375 (89.79)	2277 (89.29)	2897 (88.40)	10,032 (89.28)	161 (95.27)	1024 (87.52)
Mother resides with family	Yes	337 (12.19)	300 (11.34)	309 (12.12)	449 (13.70)	1395 (12.42)	12 (7.10)	179 (15.30)
	No	2427 (87.81)	2345 (88.66)	2241 (87.88)	2828 (86.30)	9841 (87.58)	157 (92.90)	991 (84.70)
Marital status	Single	251 (9.08)	246 (9.30)	249 (9.76)	325 (9.92)	1071 (9.53)	9 (5.33)	131 (11.20)
	Married	2409 (87.16)	2332 (88.17)	2248 (88.16)	2874 (87.70)	9863 (87.78)	145 (85.80)	996 (85.13)
	Divorced	49 (1.77)	32 (1.21)	29 (1.14)	52 (1.59)	162 (1.44)	8 (4.73)	29 (2.48)
	Widowed	54 (1.95)	28 (1.06)	17 (0.67)	22 (0.67)	121 (1.08)	7 (4.14)	12 (1.03)
	Separated	1 (0.04)	7 (0.26)	7 (0.27)	4 (0.12)	19 (0.17)	0 (0.00)	2 (0.17)
Household registration type	Urban	1084 (39.22)	1029 (38.90)	933 (36.59)	1558 (47.54)	4604 (40.98)	47 (27.81)	652 (55.73)
	Rural	1680 (60.78)	1616 (61.10)	1617 (63.41)	1719 (52.46)	6632 (59.02)	122 (72.19)	518 (44.27)
Highest educational level	PSB ^3^ (ref)	303 (10.96)	255 (9.64)	209 (8.20)	274 (8.36)	1041 (9.26)	29 (17.16)	85 (7.26)
	SPSG ^4^	1610 (58.25)	1578 (59.66)	1586 (62.20)	1721 (52.52)	6495 (57.81)	110 (65.09)	473 (40.43)
	HSTS ^5^	687 (24.86)	623 (23.55)	552 (21.65)	745 (22.73)	2607 (23.20)	22 (13.02)	357 (30.51)
	UA ^6^	164 (5.93)	189 (7.15)	203 (7.96)	537 (16.39)	1093 (9.73)	8 (4.73)	255 (21.79)
Current educational status	Yes ^7^	79 (2.86)	71 (2.68)	66 (2.59)	128 (3.91)	344 (3.06)	4 (2.37)	41 (3.50)
	No ^8^	2685 (97.14)	2574 (97.32)	2484 (97.41)	3149 (96.09)	10,892 (96.94)	165 (97.63)	1129 (96.50)
Employment status	Yes	1808 (65.41)	1852 (70.02)	1830 (71.76)	2377 (72.54)	7867 (70.02)	113 (66.86)	899 (76.84)
	No	956 (34.59)	793 (29.98)	720 (28.24)	900 (27.46)	3369 (29.98)	56 (33.14)	271 (23.16)

^1^ Participants who had previous smoking experience; ^2^ Participants who had previous drinking experience; ^3^ Primary school and below; ^4^ Secondary and primary school graduates; ^5^ High school and secondary technical school; ^6^ University or above; ^7^ Students; ^8^ Participants who were not attending school.

**Table 2:** Trends of alcohol and tobacco consumption among adult women of reproductive age by survey year.

Variable	2004	2006	2009	2011	Mantel–Haenszal *χ*^2^	*p*-Value
	*N* ^5^	*N*	*N*	*N*		
Smoking ^1^	40	47	34	48	*Z* = 0.460	0.646
Nonsmoking ^2^	2724	2598	2516	3229		
%	1.45%	1.78%	1.33%	1.46%		
Drinking ^3^	370	222	217	361	*Z* = 2.365	0.018 *
Nondrinking ^4^	2394	2423	2333	2916		
%	13.39%	8.39%	8.51%	11.02%		

^1^ Participants who had previous smoking experience; ^2^ Participants who had no previous smoking experience; ^3^ Participants with previous drinking experience; ^4^ Participants without previous drinking experience; ^5^ Number of participants; * Statistically significant (*p* < 0.05).

**Table 3:** Penalized logistic regression analysis of tobacco consumption among adult women of reproductive age (continuous variable).

Variable		Tobacco Consumption											
		2004			2006			2009			2011		
		Estimate	SE ^1^	*p*-Value	Estimate	SE	*p*-Value	Estimate	SE	*p*-Value	Estimate	SE	*p*-Value
Residence	Rural vs. Urban	−0.243	0.177	0.169	−0.191	0.165	0.245	−0.063	0.197	0.749	−0.025	0.156	0.871
Age		0.043	0.026	0.092	0.042	0.023	0.070	0.076	0.026	0.004 **	0.060	0.022	0.007 **
Father resides with family	No vs. Yes	−0.292	0.457	0.523	−0.190	0.659	0.773	1.506	0.669	0.024 *	0.278	0.412	0.500
Mother resides with family	No vs. Yes	−0.262	0.419	0.531	0.528	0.641	0.411	−0.282	0.413	0.496	0.088	0.389	0.820
Marital status	Married vs. Single	−1.313	0.545	0.016 *	−0.978	0.634	0.123	−1.516	0.536	0.005 **	−1.565	0.463	0.0007 **
	Divorced vs. Single	0.661	0.664	0.319	1.902	0.749	0.011 *	−0.743	1.167	0.525	0.103	0.632	0.871
	Widowed vs. Single	−0.247	0.747	0.741	−1.638	1.285	0.203	0.736	0.731	0.315	0.354	0.693	0.610
	Separated vs. Single	2.376	1.845	0.198	1.343	1.509	0.373	1.029	1.390	0.459	1.047	1.362	0.442
Household registration type	Rural vs. Urban	0.381	0.215	0.076	0.518	0.212	0.015 *	0.335	0.224	0.135	0.067	0.167	0.690
Highest educational level	SPSG vs. PSB	0.026	0.284	0.927	0.690	0.395	0.081	0.012	0.295	0.967	0.365	0.236	0.123
	HSTS vs. PSB	−0.696	0.358	0.052	−0.265	0.467	0.570	−0.339	0.375	0.366	−0.168	0.300	0.575
	UA vs. PSB	0.010	0.533	0.985	−1.428	0.994	0.151	0.056	0.534	0.917	−0.518	0.403	0.199
Current educational status	No vs. Yes	0.452	0.686	0.510	−0.684	0.316	0.031 *	0.115	0.679	0.865	0.042	0.409	0.918
Employment status	No vs. Yes	0.139	0.165	0.399	0.074	0.156	0.637	0.185	0.183	0.310	0.081	0.153	0.598
Alcohol consumption	Yes vs. No	0.603	0.170	0.0004 **	0.654	0.179	0.0003 **	0.871	0.195	<0.0001 **	0.633	0.162	<0.0001 **

^1^ SE, standard error; * Statistically significant (*p* < 0.05); ** Statistically significant (*p* < 0.01).

**Table 4:** Penalized logistic regression analysis of alcohol consumption among adult women of reproductive age (continuous variables).

Variable		Alcohol Consumption											
		2004			2006			2009			2011		
		Estimate	SE	*p*-Value	Estimate	SE	*p*-Value	Estimate	SE	*p*-Value	Estimate	SE	*p*-Value
Residence	Rural vs. Urban	−0.378	0.064	<0.0001 **	−0.297	0.081	0.0002 **	−0.433	0.081	<0.0001 **	−0.345	0.064	<0.0001 **
Age		−0.004	0.009	0.617	0.019	0.011	0.104	0.008	0.011	0.454	0.017	0.009	0.055
Father resides with family	No vs. Yes	0.281	0.195	0.150	0.484	0.224	0.030 *	0.027	0.197	0.892	−0.183	0.165	0.268
Mother resides with family	No vs. Yes	−0.107	0.177	0.545	−0.487	0.215	0.024 *	−0.236	0.192	0.218	0.265	0.167	0.114
Marital status	Married vs. Single	−0.143	0.486	0.769	−0.113	0.305	0.710	−0.046	0.397	0.908	0.056	0.389	0.885
	Divorced vs. Single	0.318	0.545	0.560	−0.434	0.495	0.381	−0.169	0.559	0.762	−0.096	0.503	0.848
	Widowed vs. Single	−1.049	0.654	0.109	−0.056	0.611	0.927	1.374	0.606	0.023*	−0.191	0.663	0.774
	Separated vs. Single	1.512	1.843	0.412	0.833	0.765	0.276	−1.027	1.334	0.442	−0.128	1.356	0.925
Household registration type	Rural vs. Urban	−0.064	0.072	0.380	−0.016	0.089	0.856	−0.042	0.092	0.646	0.111	0.074	0.134
Highest educational level	SPSG vs. PSB	−0.427	0.100	<0.0001 **	−0.309	0.126	0.014 *	−0.556	0.137	<0.0001 **	−0.448	0.104	<0.0001 **
	HSTS vs. PSB	0.041	0.105	0.693	0.026	0.135	0.850	0.337	0.138	0.014 *	0.154	0.111	0.167
	UA vs. PSB	0.509	0.161	0.002 *	0.819	0.182	<0.0001 **	0.761	0.179	<0.0001 **	0.718	0.126	<0.0001 **
Current educational status	No vs. Yes	0.044	0.186	0.812	−0.477	0.179	0.008 **	0.120	0.228	0.599	0.010	0.155	0.949
Employment status	No vs. Yes	−0.244	0.067	0.0002 **	−0.231	0.089	0.009 **	−0.034	0.086	0.690	−0.174	0.072	0.015 *
Tobacco consumption	Yes vs. No	0.620	0.178	0.0005 **	0.674	0.186	0.0003 **	0.894	0.208	<0.0001 **	0.647	0.171	0.0002 **

* Statistically significant (*p* < 0.05); ** Statistically significant (*p* < 0.01).

**Table 5:** Penalized logistic regression analysis of tobacco consumption among adult women of reproductive age (discontinuous variables).

Variable		Tobacco Consumption											
		2004			2006			2009			2011		
		Estimate	SE	*p*-Value	Estimate	SE	*p*-Value	Estimate	SE	*p*-Value	Estimate	SE	*p*-Value
Residence	Rural vs. Urban	−0.112	0.089	0.208	−0.194	0.164	0.236	−0.052	0.194	0.790	−0.028	0.154	0.856
Age	24–29 vs. 18–23	0.187	0.292	0.522	0.557	0.470	0.236	−0.174	0.545	0.749	−0.331	0.432	0.443
	30–44 vs. 18–23	0.738	0.247	0.003 **	0.261	0.434	0.548	0.702	0.417	0.092	0.625	0.305	0.041 *
	45–49 vs. 18–23	1.092	0.263	<0.0001 **	0.986	0.454	0.030 *	1.046	0.462	0.024 *	1.004	0.370	0.007 ^**^
Father resides with family	No vs. Yes	0.183	0.263	0.486	−0.344	0.617	0.577	1.350	0.614	0.028 *	0.160	0.394	0.685
Mother resides with family	No vs. Yes	0.042	0.241	0.861	0.521	0.605	0.389	−0.318	0.401	0.427	0.125	0.372	0.737
Marital status	Married vs. Single	−1.097	0.351	0.002 **	−0.933	0.605	0.123	−1.525	0.528	0.004 **	−1.612	0.461	0.006 **
	Divorced vs. Single	0.660	0.423	0.119	1.852	0.743	0.013 *	−0.782	1.151	0.497	0.029	0.633	0.963
	Widowed vs. Single	0.176	0.456	0.699	−1.607	1.282	0.210	0.845	0.710	0.234	0.376	0.689	0.585
	Separated vs. Single	0.046	1.172	0.969	0.797	1.501	0.596	0.947	1.298	0.466	1.028	1.367	0.452
Household registration type	Rural vs. Urban	0.312	0.104	0.003 **	0.519	0.208	0.013 *	0.333	0.223	0.134	0.088	0.167	0.596
Highest educational level	SPSG vs. PSB	0.291	0.146	0.046 *	0.732	0.388	0.059	0.001	0.293	0.996	0.366	0.236	0.122
	HSTS vs. PSB	−0.362	0.189	0.055	−0.269	0.460	0.559	−0.336	0.374	0.370	−0.125	0.300	0.676
	UA vs. PSB	−0.469	0.287	0.103	−1.444	0.968	0.136	0.032	0.523	0.951	−0.541	0.399	0.175
Current educational status	No vs. Yes	−0.070	0.246	0.777	−0.724	0.316	0.022 *	0.095	0.663	0.887	−0.055	0.409	0.894
Employment status	No vs. Yes	0.112	0.084	0.182	0.062	0.155	0.688	0.180	0.181	0.319	0.099	0.152	0.514
Alcohol consumption	Yes vs. No	0.683	0.089	<0.0001 **	0.664	0.180	0.0002 **	0.901	0.193	<0.0001 **	0.636	0.160	<0.0001 **

* Statistically significant (*p* < 0.05); ** Statistically significant (*p* < 0.01).

**Table 6:** Penalized logistic regression analysis for alcohol consumption among adult women of reproductive age (discontinuous variables).

Variable		Alcohol Consumption											
		2004			2006			2009			2011		
		Estimate	SE	*p*-Value	Estimate	SE	*p*-Value	Estimate	SE	*p*-Value	Estimate	SE	*p*-Value
Residence	Rural vs. Urban	−0.365	0.035	<0.0001 **	−0.300	0.081	0.0002 **	−0.448	0.082	<0.0001 **	−0.349	0.064	<0.0001 **
Age	24–29 vs. 18–23	−0.141	0.077	0.068	−0.356	0.201	0.076	−0.266	0.171	0.121	0.019	0.123	0.881
	30–44 vs. 18–23	0.094	0.062	0.129	0.290	0.159	0.068	−0.201	0.133	0.130	0.072	0.103	0.486
	45–49 vs. 18–23	0.187	0.074	0.012 *	0.263	0.184	0.152	0.262	0.163	0.107	0.151	0.143	0.290
Father resides with family	No vs. Yes	0.108	0.095	0.256	0.434	0.224	0.052	0.096	0.199	0.629	−0.184	0.165	0.264
Mother resides with family	No vs. Yes	−0.079	0.091	0.384	−0.465	0.215	0.030 *	−0.279	0.191	0.143	0.282	0.167	0.091
Marital status	Married vs. Single	0.017	0.174	0.921	−0.136	0.306	0.656	0.015	0.396	0.971	0.027	0.389	0.945
	Divorced vs. Single	0.093	0.231	0.689	−0.489	0.496	0.324	−0.166	0.558	0.767	−0.092	0.502	0.854
	Widowed vs. Single	−0.092	0.293	0.754	−0.053	0.610	0.930	1.381	0.606	0.023 *	−0.164	0.661	0.805
	Separated vs. Single	−0.033	0.576	0.954	0.837	0.770	0.277	−0.956	1.326	0.471	−0.107	1.352	0.937
Household registration type	Rural vs. Urban	0.007	0.040	0.871	−0.004	0.090	0.963	−0.047	0.092	0.609	0.104	0.074	0.161
Highest educational level	SPSG vs. PSB	−0.438	0.056	<0.0001 **	−0.340	0.127	0.007 **	−0.550	0.137	<0.0001 **	−0.438	0.105	<0.0001 **
	HSTS vs. PSB	0.139	0.059	0.019 *	0.033	0.136	0.807	0.298	0.139	0.032 *	0.150	0.112	0.179
	UA vs. PSB	0.649	0.076	<0.0001 **	0.826	0.182	<0.0001 **	0.777	0.179	<0.0001 **	0.683	0.126	<0.0001 **
Current educational status	No vs. Yes	−0.053	0.092	0.566	−0.456	0.181	0.012 *	0.153	0.229	0.504	−0.0009	0.157	0.996
Employment status	No vs. Yes	−0.176	0.038	<0.0001 **	−0.222	0.089	0.013 *	−0.061	0.087	0.484	−0.173	0.072	0.016 *
Tobacco consumption	Yes vs. No	0.693	0.091	<0.0001 **	0.688	0.186	0.0002 **	0.905	0.208	<0.0001 **	0.653	0.171	0.0001 **

* Statistically significant (*p* < 0.05); ** Statistically significant (*p* < 0.01).

## Supplementary Materials

The following are available online at http://www.mdpi.com/1660-4601/16/1/56/s1, Table S1: characteristics of 10,934 participants stratified by survey year (*N*, %), Table S2: penalized logistic regression analysis of tobacco consumption amongst adult women of reproductive age, Table S3: penalized logistic regression analysis of alcohol consumption amongst adult women of reproductive age.
