# Supplementary material for: Tobacco and Alcohol Consumption Rates among Chinese Women of Reproductive Age in 2004–2011: Rate and Sociodemographic Influencing Factors
Source: Int J Environ Res Public Health. 2018 Dec 26;16(1):56. doi: 10.3390/ijerph16010056 (PMC6339028; doi:10.3390/ijerph16010056)
Supplement: Supplementary file 1 [file ijerph-16-00056-s001.pdf]

Table S1. Characteristics of 10,934 participants stratified by survey year (*N*, %).

Variable		2004		2006		2009		2011		Total	
		N	%	N	%	N	%	N	%	N	%
Residence	Urban	879	33.05	832	32.27	790	31.64	1261	39.42	3762	34.41
	Rural	1781	66.95	1746	67.73	1707	68.36	1938	60.58	7172	65.59
Father resides with family	Yes	270	10.15	255	9.89	259	10.37	357	11.16	1141	10.44
	No	2390	89.85	2323	90.11	2238	89.63	2842	88.84	9793	89.56
Mother resides with family	Yes	318	11.95	279	10.82	291	11.65	415	12.97	1303	11.92
	No	2342	88.05	2299	89.18	2206	88.35	2784	87.03	9631	88.08
Marital status	Spinster	251	9.44	246	9.54	249	9.97	325	10.16	1071	9.80
	Married	2409	90.56	2332	90.46	2248	90.03	2874	89.84	9863	90.20
Household registration type	Urban	1025	38.53	989	38.36	896	35.88	1498	46.83	4408	40.31
	Rural	1635	61.47	1589	61.64	1601	64.12	1701	53.17	6526	69.69
Highest educational level	PSB[1]	286	10.75	246	9.54	207	8.29	270	8.44	1009	9.23
	SPSG[2]	1566	58.87	1553	60.24	1561	62.52	1694	52.95	6374	59.30
	HSTS[3]	653	24.55	601	23.31	533	21.35	715	22.35	2502	22.88
	UA[4]	155	5.83	178	6.90	196	7.85	520	16.26	1049	9.59
Current educational status	Yes[5]	78	2.93	70	2.72	66	2.64	126	3.94	340	3.11
	No[6]	2582	97.07	2508	97.28	2431	97.36	3073	96.06	10594	96.89
Employment status	Yes	1735	34.77	1802	69.90	1789	71.65	2325	72.68	7651	69.97
	No	925	65.23	776	30.10	708	28.35	874	27.32	3283	30.03

[1] Primary school and below; [2] Secondary and primary school graduates; [3] High school and secondary technical school; [4] University or above; [5] Students; [6] Participants who do not attend school.

Table S2. Penalised logistic regression analysis of tobacco consumption amongst adult women of reproductive age.

Variable		Tobacco consumption											
		**2004**			**2006**			**2009**			**2011**		
		Estimate	SE[1]	p-value	Estimate	SE	p-value	Estimate	SE	p-value	Estimate	SE	p-value
Residence	Rural vs. Urban	-0.2643	0.1864	0.1562	-0.1853	0.1709	0.2783	-0.0724	0.2009	0.7185	-0.0443	0.1609	0.7829
Age		0.0480	0.0274	0.0792	0.0491	0.0243	0.0428*	0.0859	0.0281	0.0022**	0.0537	0.0227*	0.0179*
Father resides with family	No vs. Yes	0.2022	0.5146	0.6944	-0.2364	0.7600	0.7557	1.3174	0.7431	0.0763	-0.0698	0.4915	0.8871
Mother resides with family	No vs. Yes	-0.5407	0.4354	0.2142	0.1744	0.7150	0.8073	0.1297	0.5108	0.7996	0.4473	0.5042	0.3750
Marital status	Married vs. Spinster	-0.0938	0.5059	0.8529	0.0602	0.5952	0.9195	-1.3067	0.4180	0.0018**	-0.8045	0.3616	0.0261*
Household registration type	Rural vs. Urban	0.3666	0.2324	0.1148	0.6040	0.2302	0.0087**	0.2631	0.2333	0.2594	-0.0480	0.1714	0.7797
	SPSG vs. PSB	-0.0149	0.3004	0.9604	0.4677	0.4003	0.2428	0.1428	0.3111	0.6462	0.3624	0.2437	0.1369
Highest educational level	HSTS vs. PSB	-0.8912	0.4172	0.0327*	-0.2245	0.4743	0.6360	-0.5705	0.4329	0.1876	-0.2385	0.3150	0.4489
	UA vs. PSB	0.2736	0.5511	0.6196	-1.0256	1.0199	0.3146	0.0581	0.5485	0.9157	-0.5785	0.4097	0.1580
Current educational status	No vs. Yes	0.3934	0.6817	0.5638	-0.6933	0.3131	0.0268*	0.1010	0.6798	0.8819	0.00247	0.4084	0.9952
Employment status	No vs. Yes	0.0399	0.1798	0.8242	0.0349	0.1619	0.8292	0.1747	0.1873	0.3510	0.00592	0.1639	0.9712
Alcohol consumption	Yes vs. No	0.6198	0.1787	0.0005**	0.6125	0.1847	0.0009**	0.8331	0.2103	<.0001**	0.6271	0.1684	0.0002**

[1] SE, standard error; * Statistically significant ($p < 0.05$); ** Statistically significant ($p < 0.01$).

Table S3. Penalised logistic regression analysis of alcohol consumption amongst adult women of reproductive age.

		Alcohol consumption											
Variable		**2004**			**2006**			**2009**			**2011**		
		Estimate	SE[1]	p-value	Estimate	SE	p-value	Estimate	SE	p-value	Estimate	SE	p-value
Residence	Rural vs. Urban	-0.3735	0.0654	<.0001**	-0.3039	0.0821	0.0002**	-0.4274	0.0824	<.0001**	-0.3559	0.0646	<.0001**
Age		-0.00453	0.00890	0.6107	0.0192	0.0116	0.0969	0.0113	0.0112	0.3127	0.0183	0.00871	0.0353*
Father resides with family	No vs. Yes	0.3262	0.2058	0.1129	0.4846	0.2392	0.0427*	-0.0783	0.2146	0.7153	-0.2415	0.1749	0.1673
Mother resides with family	No vs. Yes	-0.1742	0.1911	0.3619	-0.5582	0.2280	0.0144*	-0.1366	0.2096	0.5148	0.3319	0.1800	0.0652
Marital status	Married vs. Spinster	0.2793	0.1857	0.1326	0.1199	0.2098	0.5675	0.0268	0.1905	0.8881	-0.1658	0.1481	0.2629
Household registration type	Rural vs. Urban	-0.0425	0.0736	0.5635	0.00817	0.0906	0.9281	-0.0532	0.0930	0.5675	0.1319	0.0753	0.0799
	SPSG vs. PSB	-0.4426	0.1020	<.0001**	-0.3374	0.1276	0.0082**	-0.5695	0.1386	<.0001**	-0.4748	0.1058	<.0001**
Highest educational level	HSTS vs. PSB	0.0494	0.1073	0.6452	0.0268	0.1371	0.8448	0.3146	0.1394	0.0240*	0.1824	0.1121	0.1038
	UA vs. PSB	0.5609	0.1665	0.0008**	0.8257	0.1856	<.0001**	0.7931	0.1807	<.0001**	0.7303	0.1283	<.0001**
Current educational status	No vs. Yes	0.0408	0.1859	0.8263	-0.4776	0.1797	0.0079**	0.1213	0.2275	0.5940	-0.00628	0.1559	0.9678
Employment status	No vs. Yes	-0.2361	0.0676	0.0005**	-0.2252	0.0898	0.0121*	-0.0274	0.0874	0.7536	-0.1768	0.0727	0.0150*
Tobacco consumption	Yes vs. No	0.6226	0.1900	0.0010**	0.6213	0.1936	0.0013**	0.8178	0.2218	0.0002**	0.6323	0.1783	0.0004**

1 SE, standard error; * Statistically significant ($p < 0.05$); ** Statistically significant ($p < 0.01$).
